# A Population Model Evaluating the Consequences of the Evolution of Double-Resistance and Tradeoffs on the Benefits of Two-Drug Antibiotic Treatments

**DOI:** 10.1371/journal.pone.0086971

**Published:** 2014-01-31

**Authors:** Ellsworth M. Campbell, Lin Chao

**Affiliations:** Section of Ecology, Behavior and Evolution, Division of Biological Sciences, University of California San Diego, La Jolla, California, United States of America; University of Illinois, United States of America

## Abstract

The evolution of antibiotic resistance in microbes poses one of the greatest challenges to the management of human health. Because addressing the problem experimentally has been difficult, research on strategies to slow the evolution of resistance through the rational use of antibiotics has resorted to mathematical and computational models. However, despite many advances, several questions remain unsettled. Here we present a population model for rational antibiotic usage by adding three key features that have been overlooked: 1) the maximization of the frequency of uninfected patients in the human population rather than the minimization of antibiotic resistance in the bacterial population, 2) the use of cocktails containing antibiotic pairs, and 3) the imposition of tradeoff constraints on bacterial resistance to multiple drugs. Because of tradeoffs, bacterial resistance does not evolve directionally and the system reaches an equilibrium state. When considering the equilibrium frequency of uninfected patients, both cycling and mixing improve upon single-drug treatment strategies. Mixing outperforms optimal cycling regimens. Cocktails further improve upon aforementioned strategies. Moreover, conditions that increase the population frequency of uninfected patients also increase the recovery rate of infected individual patients. Thus, a rational strategy does not necessarily result in a tragedy of the commons because benefits to the individual patient and general public are not in conflict. Our identification of cocktails as the best strategy when tradeoffs between multiple-resistance are operating could also be extended to other host-pathogen systems. Cocktails or other multiple-drug treatments are additionally attractive because they allow re-using antibiotics whose utility has been negated by the evolution of single resistance.

## Introduction

Antibiotics and other antimicrobials have played a central role in the success of modern medicine. Through the use of such drugs we have witnessed dramatic control of bacterial and microbial pathogens. However, unlike many other medical practices, the deployment of antibiotics creates problems for its own sustainability. Because the target is a quickly reproducing organism, the use of antibiotics initiates a process of natural selection that counters the efficacy of the drugs on short timescales. The evolution of resistance in microbes threatens to undermine the many health benefits that we have come to take for granted [1]–[4].

Many strategies have been proposed to control the evolution of drug resistance through the rational use of antibiotics. Some simple ones are easily justified. Pathogens should be screened whenever possible to ensure that antibiotics are targeted against sensitive bacteria [2]. Research should be supported to discover new antibiotics more rapidly than pathogens are able to evolve resistance [5]. However, given the slow pace of drug development, there has also been the desire to consider more complex strategies that stop, or minimally, slow the evolution of resistance [6]. For example, is the coordinated use of two drugs better than random administration? Antibiotics have been cycled and argued to be an improvement over the status quo [7]. Switching from cephalosporin to carbapenem over a period of one year increased the frequency of resistance to carbapenem in a hospital while the level of cephalosporin resistance was reduced [8]. Long-term antibiotic switching deployments are necessary to determine whether resistance reductions are sustainable. Multi-drug cocktails have been deployed with success against cancer, HIV, tuberculosis and agricultural pathogens [9]–[11]. However, the mechanisms responsible for these successes and their long-term consequences are not well understood. Are multi-drug cocktails effective because they are analogous to a two-front offensive on a pathogen? Can patients be effectively treated with low-dose multi-drug cocktails? Are multi-drug cocktails capable of a sustained reduction in the frequency or level of antibiotic resistance?

Confounding these issues is the perception that the use of antibiotics necessarily introduces a tragedy of the commons dilemma [12], [13]. While an individual is helped by antibiotic treatment, the future public is hurt because the treatment naturally selects for the evolution of more prevalent and increased resistance in the environment. Limiting antibiotic use can control the evolution of resistance, but how such a policy translates to improved health outcomes remains unclear. The ultimate goal of sustainable management of limiting antibiotic resources should be the treatment and healthful recovery of the patient, not necessarily a reduction in prevalence of antibiotic resistance. Otherwise, the optimal strategy for antibiotic use is trivial: a global ban.

Increased microbial sensitivity to chemotherapeutic agents when used in combination, rather than in isolation, was first documented by a seminal study that sought to classify antibiotics by measuring cross-resistance [14]. Further inquiry showed that the minimum drug concentration required to inhibit microbial growth can be reduced in multiple clinically-isolated drug-resistant pathogens with the addition of sodium clavulanate [15]. Indeed, in some cases minimum inhibitory concentrations (MIC) of clinically relevant pathogens decreased by four orders of magnitude [16]. These early studies show that the evolution of antibiotic resistance can be managed, but not reversed. However, later research focused illustrates that the combination of tetracycline and fusaric acid can selectively enrich tetracycline-sensitive mutants from clonal populations of tetracycline-resistant bacteria [17]. Mechanistically, this occurs because the efflux pump responsible for tetracycline resistance (*TetA*) is hindered in the presence of fusaric acid [18]. These studies serve as a proof-of-concept that resistance characters harbored by human pathogens can be controlled in the lab. This counterbalancing force to the evolution of antibiotic resistance is also echoed in nature; common soil microbes are capable of producing fusaric acid in quantities experimentally shown to selectively enrich sensitive variants in their natural environment [15], [16], [19]. Indeed, a recent article reviews a plethora of naturally occurring mechanisms that have evolved to counter the evolution of antibiotic resistance [20].

Resolving these questions and issues has been difficult because evolution in a clinical setting is not readily amenable to controlled experimental studies. As a result, research in the field has relied heavily on mathematical and computational models to examine the efficacy of antibiotic strategies [21]–[27]. A surprising outcome is that cycling is less effective at minimizing resistance than mixing (two antibiotics used simultaneously) [21], [22]. The outcome is explained by the fact that more infected patients are cured in mixing at any point in time [8]. By this argument, mixing three or more antibiotics should be even more beneficial. All these models allowed full single-resistance but ignored the possibility of constraints or tradeoffs on double- or multiple-resistance to more than one drug. For example, a tradeoff would emerge if mutations increasing resistance to drug A express negative pleiotropic effects that decrease resistance to drug B [14], [29]–[31]. Could such pleiotropy have accelerated the reported decline in above noted cephalosporin resistance [8]?

Pleiotropic mutations and tradeoffs have historically been of interest to evolutionary biologists because they can constrain the evolutionary response to natural selection. If an organism is responding to two opposing selective forces, the two resulting adaptive responses could be slowed or curtailed by tradeoffs. While tradeoffs may be undesirable for maximizing adaptations, they could be desirable if the goal is to prevent adaptation, such as in stopping the evolution of antibiotic resistance. Determining whether a combination therapy is capable of both treating a drug-resistant infection and modulating the level of resistance is a non-trivial task [32]. Most drug-pairings have additive antimicrobial effects, but some act synergistically to produce more powerful effects than their constituent parts would suggest [33]. Experimental evidence suggests that synergistic drug pairings may increase the strength and rate at which single and multiple drug resistance evolves under treatment [34]–[36]. However, antagonistic and suppressive drug-pairings may be capable of treating resistant-infections while selectively enriching susceptible variants [37]. For example, when protein and DNA synthesis inhibitors are used in concert, sensitive variants outcompete their drug-resistant counterparts. Under this suppressive combination treatment, drug-resistant mutants are unable to maintain optimal regulation of ribosomal genes and thus incur substantial metabolic costs [28]. Mechanisms that give rise to these complex interactions are not well understood *in vitro* and have not, to our knowledge, been studied in clinical trials. Can cocktails be used safely and effectively to treat hospital-borne drug-resistant infections? Perhaps more importantly, can a pathogen’s ability to evolve high-level drug resistance be constrained by careful selection of drug cocktails that exploit evolutionary tradeoffs associated with resistance acquisition? If shown to be valid, two- or multiple-drug treatments exploiting tradeoffs become increasingly attractive because they give new life to old antibiotics that have been rendered useless by the evolution of single-resistance [30]. Indeed, there is evidence to suggest that chemical compounds, previously disregarded as ineffective when used in isolation, may be therapeutically effective in combination [6].

We have developed and analyzed a model that explores the consequences of tradeoffs on two-drug strategies by modifying the model of Bergstrom *et al.* [21]. To describe the joint effect of two drugs in a cocktail, we added to their model the pharmacodynamic equations of Regoes *et al.* [38]. Pleiotropy was introduced through a new parameter in the pharmacodynamic equations. Although double positive epistatic mutations can also influence the evolution of resistance, they are not included in our model because we consider the effects of single mutations as they arise. The phenotype of the single mutation could be influenced by its epistatic interactions with previous mutations, but what matters is phenotypically expressed double-resistance as represented by the tradeoff. The model was analyzed by tracking the frequency of patients infected with resistant bacteria, but unlike previous studies we sought conditions that maximized the frequency of uninfected patients, rather than ones that minimized antibiotic resistance. Following the analysis of Bergstrom *et al.*, we focused on the general mathematical properties of the dynamical system, rather than developing detailed quantitative predictions. Thus, we employed parameter values in the range previously used by Bergstrom *et al.* and Regoes *et al.*, and examined the resulting ecological and evolutionary processes at work in the system.

## Model

The model of Bergstrom *et al.* consists of four differential equations that describe an open hospital system in which patients are treated with antibiotics for a nosocomial infection. The patient population in their model is represented by four frequency groups *X* (uninfected), *S* (infected with sensitive bacteria), *R*_1_ (infected with bacteria resistant to antibiotic A), and *R*_2_ (infected with bacteria resistant to antibiotic B). *X* patients become infected at a rate *β* by contact with *S, R*_1_ and *R*_2_. Superinfection is also allowed at a rate *σβ* in which bacteria from *S* can colonize and take over *R*_1_ and *R*_2_ patients. The takeover of *S* by *R*_1_ and *R*_2_ bacteria is assumed not to occur because resistant bacteria are inferior competitors due to a cost *c*. Infected patients are cured of their bacteria by a clearance rate *γ*, which can be augmented by an amount *τ* with antibiotic treatment if the bacteria are sensitive. The system is open and therefore *X*, *S, R*_1_, and *R*_2_ patients enter and leave the system at set rates. The population growth rate of the four groups is described as a set of four differential equations that are coupled through infection, superinfection, clearance, immigration and emigration.

Our new model consists of the five differential equations
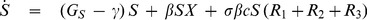
(1)

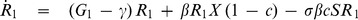
(2)

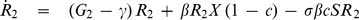
(3)

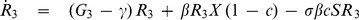
(4)

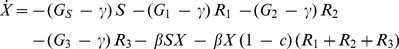
(5)where the raised dot denotes a time derivative (e.g. 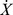 =  d*X*/dt); and *X*, *S, R*_1_, *R*_2_, *β, σ*, *c* and *γ* are as defined by Bergstrom *et al.* It should be noted that our model is constrained to situations where there exists a cost of resistance acquisition, such as large plasmid-borne resistance genes. While Equations 1–5 preserves the main features of the Berstrom *et al.* model, two major changes were incorporated.

First, we made our model a closed system because we wanted to examine the consequences of drug management without the constraints of immigration and emigration. For example, if immigration rates are extremely high, they can dominate the outcome of the model and negate the effects of drug management. The effects of drug management only emerges as immigration is sufficiently reduced, in which case it is just as reasonable to remove immigration and emigration completely. Thus, our model can be construed to represent hospitals in which the immigration and emigration are sufficiently low as to not influence the qualitative results.

The second modification was made to allow the modeling of both a resistance tradeoff between drugs A and B and the use of cocktails with the two drugs. To account for resistance tradeoff, we introduced *R*_3_ patients (Equation 4), who are infected with bacteria resistant to both drugs A and B. To incorporate a cocktail, we replaced Bergstom *et al.*’s drug-clearance rate *τ*, which is a constant independent of drug concentration, with a function *G* that varies with the concentration of drugs A and B. Because *G* should be proportional to bacterial growth rate, we chose to model it with Regoes et al.’s pharmacodynamic equation of bacterial net growth. However, because Regoes et al. developed their equation to describe growth as a function of the minimal inhibitory concentration (MIC) of only one antibiotic, we added new MIC parameters, *MICA* and *MICB*, for drugs A and B. Additionally, we developed two pharmacodynamic equations, termed *combined* and *separate*, to model cocktails. *Separate* is thusly named because the its constituent antibiotics are additive and thus held separate in the equation. The constituent antibiotics of the *combined* cocktail are not additive, but suppressive, and are therefore combined in the equation. Thus, 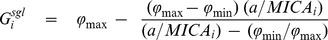
(6)

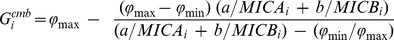
(7)

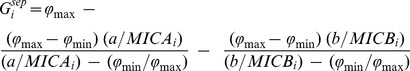
(8)where the subscript *i* denotes the four bacterial strains *S, R*_1_, *R*_2_, and *R*_3_ (see Equations 1–4); *a* and *b* are concentrations of drugs A and B; *φ_max_* is the maximal growth rate in the absence of a drug; *φ_min_* is the minimal growth rate in the presence of a drug at high dosage; and *sgl*, *cmb*, and *sep* denote single, *combined* and *separate*. The Hill coefficient *k* of Regoes *et al.*’s equation is assumed to equal one and is therefore not included in Equations 6–8. Equation 6, which is the equation of Regoes *et al.*, was used when we modeled use of only one antibiotic. A detailed interpretation of the difference between *combined* and *separate* is presented in the **Discussion**.

**Figure 1:**
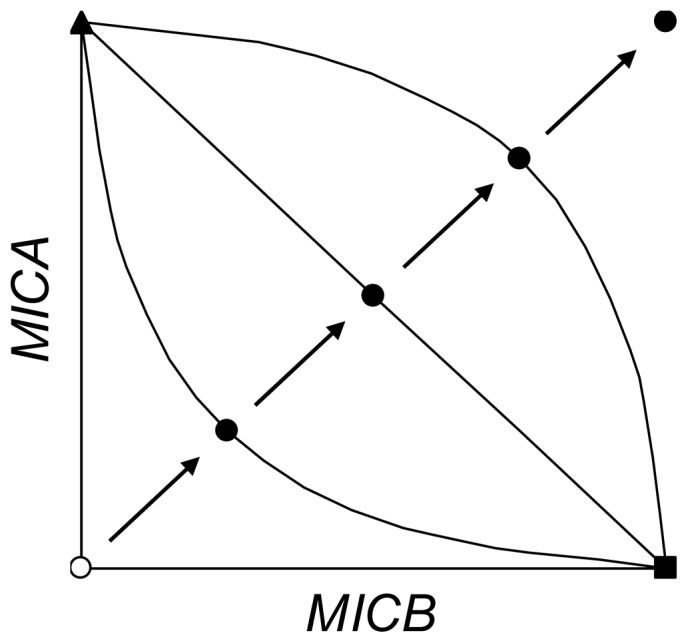
Tradeoffs in resistance to two antibiotics. Plot represents resistance as the minimal inhibitory concentration (*MICA and MICB*) to the pair of drugs A and B. The three resistant bacteria *R*_1_ (▴), *R*_2_ (▪), and *R*_3_ (•) are depicted. *R*_1_ is resistant only to drug A, and *R*_2_ only to drug B. *R*_3_ is double-resistant and four representatives are shown to denote different levels of tradeoffs. The *MICA*_3_ and *MICB*_3_ values for *R*_3_ were derived as *MICA*_3_ =  (1–*ω*)·*MICA*_1_ and *MICB*_3_ =  (1–*ω*)·*MICB*_2_, where *MICA*_1_ and *MICB*_2_ are the minimal inhibitory concentrations of *R*_1_ and *R*_2_ against drugs A and B, *ω* is the tradeoff parameter, and 0≤*ω*≤1. With the highest tradeoff constraint and *ω* = 1, *MICA*_3_ and *MICB*_3_ equal zero and are same as the *MICA_S_* and *MICB_S_* values for sensitive *S* bacteria (○). As *ω* is decreased from 1.0, 0.75, 0.50, 0.25 to 0, the progression (→) represents the double-resistant *R*_3_ experiencing less and less tradeoff. The solid lines (─) through three of the *R*_3_ points represent families of mutants that connect *R*_1_ and *R*_2_ and have the same approximate level of tradeoff. If *ω* = 0.5, the tradeoff is linear. Our examination of tradeoffs uses *R*_3_ as the representative of the family.

The strength of the tradeoff between the resistance of *R*_3_ bacteria to drugs A and B was quantified by a new parameter 0 ≤ *ω* ≤ 1 (Figure 1). A value of *ω* = ½ corresponds to a mutation with a linear tradeoff relative to *R*_1_ and *R*_2_ bacteria. For example, if *R*_1_ and *R*_2_ have, respectively, [*MICA*, *MICB*] values of [240, 0] and [0, 240], a linear tradeoff gives *R*_3_ bacteria MIC values of [(1– *ω*)·240, (1– *ω*)·240] or [120, 120] with *ω* = ½. A pleiotropic mutation that produces greater than linear tradeoff has a *ω* > ½. For example, if *ω* = ¾, *R*_3_ bacteria have [*MICA*_3_, *MICB*_3_] of [(1– ¾)·240, (1– ¾)·240] or [60, 60]. Conversely, a pleiotropic mutation that produces less than linear tradeoff has a *ω*<½. A value of *ω* = 0 denotes *R*_3_ bacteria that have [*MICA*_3_, *MICB*_3_] of [240, 240] and that are able to resist fully both drugs A and B without any tradeoff.

A more intuitive summary of our model is provided by noting that all terms that contain either *γ*, *σβ*, or *β* (without *σ*) in Equations 1–5 denote gains and losses due to clearance, superinfection, and infection, respectively. Gains and losses are inherent because any positive term in one equation surfaces as a negative term in another. For example, the term (*G_i_* – *γ*) is always negative in our model because *G_i_* ≤ 0 with our parameter values (see below). Thus, (*G_i_* – *γ*) in Equations 1–4 quantifies the curing and loss of infected patients in the *S, R*_1_, *R*_2_, and *R*_3_ populations, and – (*G_i_* – *γ*) in Equation 5 represents the addition of cured and uninfected patients to the *X* population.

## Analytical Solutions

**Figure 2:**
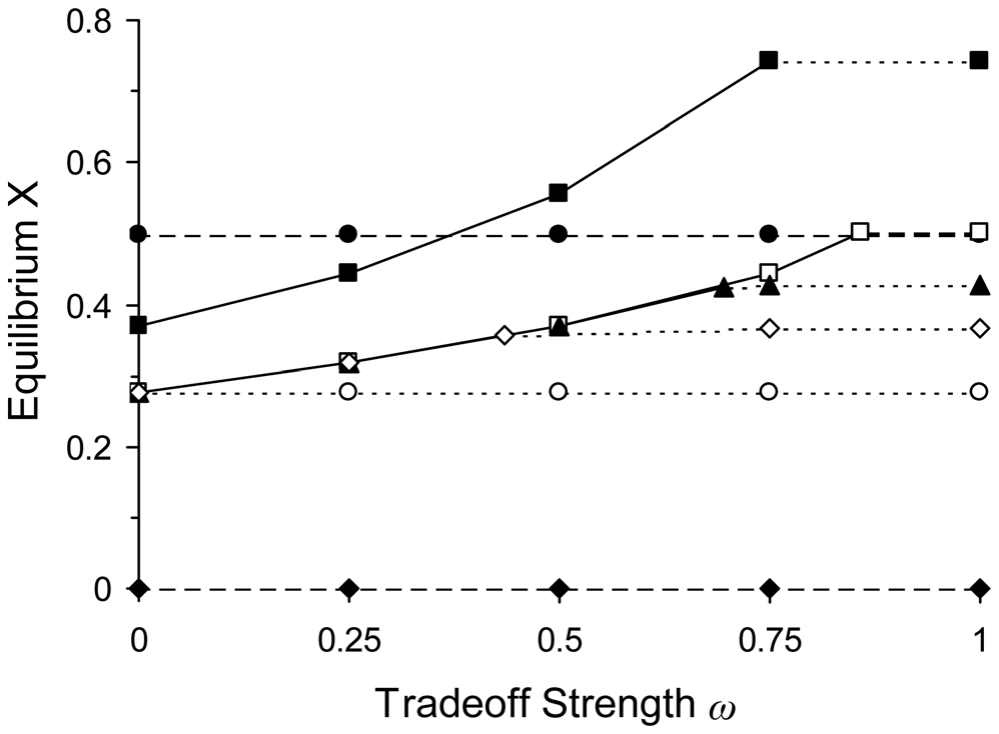
Effect of tradeoff strength on equilibrium *X* for different antibiotic treatments. Tradeoff strength is measured as the parameter *ω* (Figure 1). The equilibrium frequency of uninfected patients *Xˆ* was determined by running simulations of the model until all frequencies were stable. In cases where the values fluctuated, the equilibrium frequency was the average for the cycles. All simulations were run with parameter values of *MICA*_1_ = *MICB*_2_ = 240; *MICA_S_* = *MICB_S_* = *MICA*_2_ = *MICB*_1_  = 0; *MICA*_2_ and *MICB*_3_ as defined in Figure 1; *φ*_max_ = 0.25; *φ*_min_ = –0.25; *c* = 0.1; *σ* = 0.25; *β*  = 1.0; and *γ* = 0.25. These values are in the range of the numbers used by Bergstrom *et al.* and Regoes *et al.* During simulations, an MIC with a value of zero was reset to 0.000001 to avoid division by zero in Equations 6–8. Treatments: NONE ( ); CONTROL (•); SINGLE (○); CYCLING (*π* = 50,▴); MIXING (*π* = 50, ▴); Combined COCKTAIL (□); Separate COCKTAIL (▪). Our analysis included values of *π* greater and smaller than 50 for CYCLING and MIXING, but the data are not presented to avoid crowding the figure. We note here that if *π* = 1, *Xˆ* values in both treatments became indistinguishable from those in MIXING with *π* = 50 and for all sampled values of *ω*. If π was increased above 50, *Xˆ* values in CYCLING converged downwards to match those in SINGLE, while in MIXING they decreased to match those in CYCLING (*π* = 50). The largest value examined was *π* = 50,000. The matching of MIXING (*π* = 50,000) and CYCLING (*π* = 50) is coincidental, as the value of *π* = 50 was chosen arbitrarily. The composition of the infected population (*S*+*R*_1_+*R*_2_+*R*_3_) is shown in the graph by using the line pattern to represent the most common bacterium in the population; *S* (– – –); *R*_1_ and/or *R*_2_ (_· · ·_); *R*_3_ (—). For example, in Combined COCKTAIL (□), the most common bacterium is *R*_3_ from 0≤*ω*<0.86, but changes to *S* from 0.86≤*ω*≤1.

Using the numerical solutions for the computer simulations as a guide, we were able to obtain analytical solutions of equilibrium frequency of uninfected patients 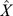 for NONE, CONTROL, SINGLE, and COCKTAIL. Solutions were not found for CYCLING and MIXING because the systems fluctuated over time. Thresholds for transitions of the dominant patient type were also derived for COCKTAIL. Analytical solutions were evaluated with the parameters (hereafter the standard values) presented in Figures 1 and 2: *γ* = 0.25; *β* = 1; *c* = 0.1; *φ*_max_ = 0.25; *φ*_min_ = – 0.25; *MICA_s_* = *MICB_s_* = *MICA*_2_ = *MICB*_1_ = 0; *MICA*_1_ = *MICB*_2_ = 240; *MICA*_3_ =  (1 – *ω*) *MICA*_1_; and *MICB*_3_ =  (1 – *ω*) *MICB*_2_. All comparisons between the numerical (Figure 2) and analytical solutions yielded matching results. Because we often relied on the numerical simulations to eliminate variables, the analytical solutions are valid only in the neighborhood of the parameter values and equilibria we examined in our study. Nonetheless, besides providing a more detailed interpretation of our results, the analytical solutions verify that the numerical solutions were valid and accurate for the reported conditions.

## Results

Antibiotic deployment strategies (NONE, SINGLE, MIXING, CYCLING, COCKTAIL, and CONTROL) are evaluated with respect to variable multi-drug resistance tradeoff (0<*ω*<1) conditions. Strategy outcomes are compared according to the value of populations *X*, *S, R*_1_, *R*_2_ and *R*_3_ at equilibrium. Excluding NONE as a no-treatment baseline, the total amount of drugs administered under each strategy remains constant at 240 dosage units (see Tables S1–S3). SINGLE strategy dictates that all patients receive the same drug (e.g., drugs A:B at dosage 240:0 and *vice versa*). While MIXING assumes that 50% of patients receive drugs A:B at 240:0 (and the other 50% of patients receive drugs A:B at 0:240). Under MIXING, each half of the population alternates A:B dosage (240:0 → 0:240, and *vice versa*) every time period of length *π.* CYCLING represents a scenario where all patients receive a single drug for a time period of length *π*, after each period all patients receive the alternate drug. COCKTAIL dictates that all patients receive both drugs A and B simultaneously, but each at reduced dosage (drugs A:B, 120:120). Finally, CONTROL represents the optimal outcome when *resistance cannot evolve* and an antibiotic successfully controls the infection. In CONTROL, the frequency of *R*_1_, *R*_2_ and *R*_3_ were set and maintained at zero and a single antibiotic was administered to infected patients (drugs A:B, 240:0).

We analyzed our model by solving the equations numerically over time by the Runge-Kutta 4th Order method in MatLab until equilibria were obtained. Analytical solutions for equilibria were obtained for NONE, CONTROL, SINGLE, and COCKTAIL (see File S1). We report in **Results** only the numerical solutions to provide data that were collected by a single procedure. Whenever analytical solutions were possible, they matched the equivalent numerical solutions. For static treatment regimens, numerical solutions were collected 100,000 timesteps after convergence to the sixteenth decimal. COCKTAIL treatment regimens impose drug clearance on all variants and therefore converged in <200 timesteps. In contrast, SINGLE requires 10,000 timesteps to converge to the sixteenth decimal. SINGLE requires more time to reach equilibrium because, at any given moment, at least one variant can resist drug treatment and can only be cleared by the patient’s immune response. Given fluctuation of population frequencies, analytical solutions were not obtained for CYCLING and MIXING. For these treatment regimens, numerical values were collected after cyclical upper and lower bounds converged to the sixteenth decimal. After which point, numerical solutions were collected and averaged for each successive timestep for 1000 periods, *π.*

For single-drug strategies the effect of tradeoff, *ω,* on the equilibrium frequency of uninfected individuals has a lower bound set by the NONE strategy (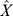 =  0%) and an upper bound set by the CONTROL scenario (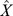 =  50%). In the absence of antibiotics (NONE), the susceptible variant is able to infect all patients in the system. In the absence of resistant mutants, half of the patients remain uninfected. Given that *R*_1_, *R*_2,_ and *R*_3_ were rare or non-existent in NONE and CONTROL, the results were not affected by variation in *ω*. After inclusion of *R*_1_, *R*_2_ and *R*_3_ under the SINGLE regimen, 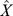
decreased by nearly half (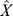 =  27.8%). 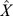
is not affected by changes in multi-drug resistance tradeoff, *ω*, because the single resistant mutants (*R*_1_, *R*_2_) outcompete the multi-resistant variant (*R*_3_) when only one drug is present in the environment. These results serve as a baseline with which to compare two-drug implementations that exploit variation in space (MIXING), time (CYCLING), or dose concurrency (COCKTAIL).

CYCLING and MIXING treatment regimens responded to changes in tradeoff strength, *ω.* In both cases, 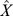 was equivalent to the SINGLE treatment regimen when the multi-resistant variant is super-resistance, *ω* = 0. As *ω* was elevated, 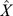 increased, but never converged with the CONTROL scenario wherein drug resistance cannot evolve. At *π* = 50 and *ω*>0, MIXING always outperformed CYCLING (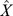 =  42.7% and 36.5%, respectively; *ω* = 1). As *π* decreases from 50 to 1, 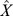 values remain unchanged for MIXING over all examined tradeoffs, (0<*ω*<1). However, as *π* decreases from 50 to 1, 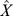
values achieved by the CYCLING regimen approach that of MIXING. Put another way, as the rate of antibiotic cycling increases, the difference between CYCLING and MIXING become negligible. If *π* is increased beyond 50, CYCLING and MIXING responded differently. While CYCLING converged downwards onto SINGLE as *π* is increased, MIXING decreased to equilibrium above SINGLE.

We interpret the dependency of 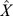
on *ω* in CYCLING and MIXING to result from the transformation of *R*_3_ into super-sensitive or super-resistant bacteria as a consequence of the tradeoff. When *ω* equals zero or is low, *R*_3_ bacteria are super-resistant and the system becomes effectively equivalent to having one resistant bacterium and one antibiotic (as in SINGLE). On the other hand, *R*_3_ bacteria become super-sensitive when *ω* equals one or is high. As a result, *R*_3_ are eliminated by the antibiotics and *R*_1_ and *R*_2_ become common. However, because *R*_1_ and *R*_2_ are resistant to only one antibiotic, they are held in check more easily and 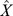 increases as a result. This interpretation was verified by tracking the most common bacterial type (S, *R*_1_, *R*_2_ or *R*_3_) in the treatments. In both CYCLING and MIXING, the dominant bacterial type switched from *R*_3_ to a mixture of *R*_1_ and *R*_2_ as *ω* was increased beyond threshold values of *ω* = 0.44 and 0.70, respectively.

The value of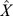 also rose as values of *ω* were increased in the *combined* and *separate* COCKTAIL treatments (Figure 2). However, the gains were greater than in CYCLING and MIXING, and 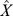 increased to 50% and 74.1%, respectively, as *ω* approached a value of one. These values of 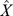
come close or exceed the 50% that we observed before the evolution of resistance (CONTROL; Figure 2). In *combined* COCKTAIL, there was no advantage for *ω* = 0 because *R*_3_, as a super-resistant bacterium, was again the most common. As *ω* was increased, *R*_3_ was replaced at a threshold of *ω* = 0.86 and *S* became the most common bacterial type. In *separate* COCKTAIL, there was an advantage when *ω* = 0 and 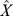 attained a value of 37.0%, which easily beat the 27.8% baseline of SINGLE, despite the fact that *R*_3_ dominates. As *ω* was increased, *R*_3_ was replaced at a threshold of 0.75 by *R*_1_ and *R*_2_. The reason for the dominance of *S* bacteria and the greater than baseline value of 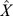
in the COCKTAIL treatments are addressed in the **Discussion**.

**Figure 3:**
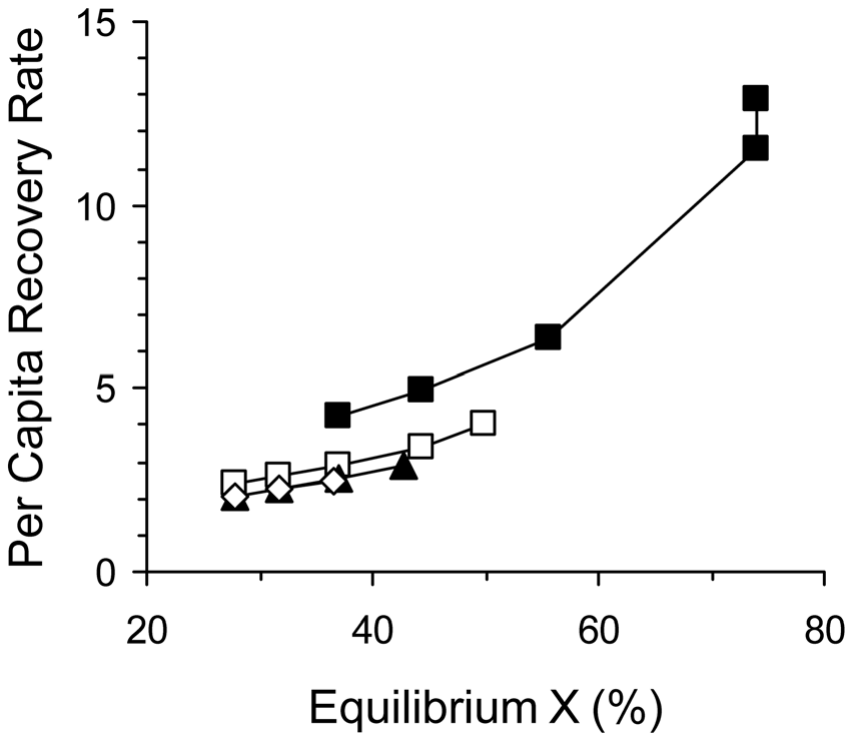
Positive correlation between *X* and per capita recovery rate for double- drug treatments at equilibrium. The equilibrium value *Xˆ* determined as in Figure 2. The per capita recovery rate at equilibrium was measured by obtaining first the population recovery rate from Equation 5 as *z* = – (*G_S_* –*γ*) _·_ *S* – (*G_1_* –*γ*) *R*_1_ – (*G_2_* –*γ*) *R*_2_ – (*G_2_* –*γ*) *R*_3_, where *S, R*_1,_ *R*_2,_ & *R*_3_ are equilibrium values. The per capita recovery rate was then derived as *z/*(*S*+*R*_1_+*R*_2_+*R*_3_). The per capita recovery rate is positive because (*G_i_* –*γ*) ≤ 0 for the parameter values in our model (see Figure 2). Treatments: CYCL ING (*π* = 50, ▴); MIXING (*π* = 50, ▴); Combined COCKTAIL (□); Separate COCKTAIL (▪);

An issue in the design of strategies for antibiotic use is whether benefits to the individual and the population are in conflict. Our maximization of 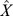 to assess the efficacy of different antibiotic treatments assumes that a benefit to the patient population is paramount. To determine whether a benefit to the population is beneficial or detrimental to individual patients, we also monitored the equilibrium *per capita recovery rate* of patients as a function of 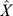 (Figure 3). In all of the treatment with two antibiotics (COCKTAIL, MIXING, and CYCLING), 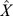 and the per capita recovery rate were positively correlated. Thus, at least from this perspective, there is no conflict between benefits to the individual and population in our model at equilibrium. Additionally, for the same values of 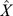, *separate* COCKTAIL yielded the highest *per capita recovery rates* when compared to all the other two-drug treatments.

## Discussion

Genetic tradeoffs, also known as developmental constraints, have long been regarded as barriers to evolution by natural selection [39]–[41]. If two phenotypic traits are linked by tradeoffs, selection cannot easily maximize fitness at both. Tradeoffs are considered undesirable because they can slow and even possibly stop adaptation. Our work attempts to turn the tables and asks if tradeoffs could be made useful for slowing or stopping the evolution of adaptations that are detrimental to us, e.g. antibiotic resistance in bacteria. If two antibiotic drugs are used against an infectious bacterium, natural selection should favor increased resistance to both drugs. If resistance is constrained by tradeoffs, conditions could arise in which the patient population would benefit. To explore this possibility, we developed and analyzed a population model with clinical patients experiencing infections by a hospital-acquired pathogen. Two drugs were deployed and the tradeoff was represented by three resistant bacteria, *R*_1_, *R*_2_ and *R*_3_. While *R*_1_ and *R*_2_ were resistant only to one drug (A and B, respectively), *R*_3_ was double resistant. However, the resistance of *R*_3_ was subject to a tradeoff that was quantified by a parameter *ω*, which ranged from zero to one to represent increasing strength of the constraint (Figure 1).

We examined first the NONE, CONTROL and SINGLE treatments. The resulting equilibrium frequency of uninfected patients (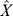; Figure 2) showed clearly the advantage of antibiotics when resistance was absent (or has not yet evolved). Without the application of antibiotics in NONE, 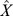 =  0% and the entire population was infected. Without the presence of resistance in CONTROL, the use of antibiotics controlled the infection and 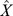 increased to 50%. With the presence of resistant mutants in SINGLE, 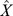 was reduced 27.8% when only one antibiotic is used. NONE, CONTROL, and SINGLE correspond to the baselines to which all other treatments have to be compared. NONE represents the condition when patients are infected by a bacterium before the advent of antibiotics. CONTROL demonstrates the benefits an antibiotic provides before the evolution of resistance. SINGLE is the scenario when resistance evolves and undermines the efficacy of the antibiotic. Thus, SINGLE serves as the minimum baseline that any two-drug treatment must surpass to be advantageous while CONTROL sets the baseline to which any resulting advantage must be compared.

The two-drug treatments CYCLING and MIXING generated similar outcomes (Figure 2). In both cases, 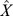
could be higher than the level in SINGLE, but never more than that in CONTROL, and the advantage depended on *ω*. When *ω* = 0 (no tradeoff), there was no advantage because *R*_3_ is resistant to both drugs (super-resistant). At the other extreme of *ω* = 1 (maximal tradeoff) the advantage was greatest because the two resistances in *R*_3_ interfere with each other and the bacteria are rendered super-sensitive. The response of CYCLING and MIXING to *ω* depend on the length of the time period *π*. For small values (*π* = 1) 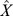 for the two treatments converged to the same value. The convergence results because cycling two antibiotics with a vanishingly small period is effectively equivalent to mixing [21]. While 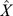 for these strategies converge to the same value, CYCLING requires more time to reach equilibrium than MIXING. For intermediate values (*π* = 50), 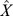 in MIXING achieved higher values than in CYCLING. For longer values (*π* >> 50), 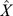 in CYCLING converged downwards to the values in SINGLE, while 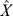 in MIXING converged downwards to an equilibrium above SINGLE. CYCLING converges to SINGLE because as *π* becomes infinitely long, the bacteria in CYCLING encounter effectively only one antibiotic over time. 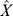 in MIXING is always higher at both intermediate and higher values of *π* because of the presence of two antibiotics at all times [8]. As a result, *R*_1_ and *R*_2_ bacteria can encounter the drug to which they are sensitive (B and A, respectively) through infection or superinfection, even as *π* becomes infinitely long in MIXING.

Bergstrom *et al.*’s previous results demonstrating the superiority of mixing over cycling are replicated by our outcomes for *ω* = 1. Because Bergstrom *et al.* did not model double-resistant mutants, we reproduced their conditions by letting *ω* = 1 and making *R*_3_ super-sensitive. Thus, our model extends the model of Bergstrom *et al.* by introducing *R*_3_ and shows that CYCLING and MIXING can be more advantageous than using only one antibiotic (as in SINGLE), but only when strong or moderate tradeoffs *and* a cost, *c,* of resistance are present. With weak or no tradeoff, *R*_3_ is able to dominate and render CYCLING and MIXING no better than using only one antibiotic. If the cost of resistance is negligible, *S* cannot compete with resistant mutants in cases of superinfection.

To model COCKTAIL treatments, we formulated the *combined* and *separate* (Equations 7 and 8) to describe the simultaneous effects of two drugs on bacteria. Although the advantage of the two-drug COCKTAIL treatments again increased with *ω*, their behaviors were qualitatively different (Figure 2). In *combined* COCKTAIL, 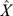
equaled the baseline of 27.8% in SINGLE when *ω* = 0, but increased to 50% when *ω* = 1. At higher values of *ω*, the larger 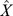 results because *R*_3_ is unable to persist and is replaced by the sensitive bacteria *S*. *Separate* COCKTAIL yielded even larger 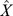
values. At *ω* = 0, 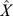 was 37.0% and considerably higher than the 27.8% baseline of SINGLE. At *ω* = 1, 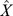
rose to 74.1%, which is better than the high frequencies of uninfected patients that were generated by CYCLING, MIXING and *combined* COCKTAIL. Most surprisingly, the highest values of 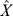
in *separate* COCKTAIL surpassed even the frequencies we had observed in CONTROL. Thus, the two-drug *combined* COCKTAIL treatment, even when facing single- and double-resistance, can outperform a single drug treatment in the absence of resistance (cf. CONTROL).

The distinct behaviors of our COCKTAIL treatments result from our formulation of Equations 7 and 8. The presence of only one negative term in Equation 7 explains why *S* dominates at low values of *ω* in the *combined* COCKTAIL treatments, despite the presence of antibiotics. With only one negative term, *S* and resistant bacteria are all cleared at approximately the same rate as *ω* approaches zero. Thus, *S* dominates because it does not pay the cost *c* of resistance while all else is approximately equal (see Equations 1–5). It is important to note that selective enrichment of *S* under *combined* COCKTAIL treatment requires resistant mutants to pay a cost *c* and experience a powerful trade as *ω* approaches zero. Alternatively, the advantage of *separate* COCKTAIL over all values of *ω* results from the presence of two negative terms in equation 8. With two terms detracting from their growth rate, the resistant bacteria are doubly hurt and 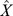 increases beyond the values in SINGLE, CYCLING, MIXING and CONTROL. The effect is sufficiently strong even when *R*_3_ dominates and *ω* = 0 or intermediate.

The interplay between the tradeoff strength *ω* and the rise of *R*_3_ governs the outcomes of all the two-drug treatments we examined. If *ω* is high or intermediate, *R*_3_ is held down and an advantage emerges over using a single antibiotic for CYCLING, MIXING, *separate* COCKTAIL, and *combined* COCKTAIL (in increasing rank). It is noteworthy that *separate* COCKTAIL achieved an advantage over CONTROL even when *ω* = 0.5 (Figure 2). While an extreme tradeoff of *ω* = 1 may be biologically unrealistic, a linear one of *ω* = 0.5 may not. If our results can be replicated *in vivo*, antibiotic cocktails will provide a powerful tool to control the evolution of bacterial resistance while maximizing the health of a patient population. Cocktails are additionally desirable because their administration would be free of the complex timing required by cycling and mixing strategies.

It is also noteworthy that the *per capita recovery rate* correlated positively with 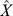 in our model for CYCLING, MIXING, and COCKTAIL (Figure 3). Moreover, *separate* COCKTAIL again stood out as a better treatment when individual and public welfare are compared. For a given 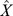
(e.g. 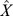 =  40%; Figure 3), *per capita recovery rate* was higher in *separate* COCKTAIL relative to the other two-drug treatments. These results show that it is not necessarily valid that the general public must be hurt by the evolution of resistance whenever a sickened individual patient is helped by antibiotics [12], [13]. It strikes us that the conflict between the individual and the public may not emerge with two-drug cocktails that effectively exploit tradeoffs. The tragedy of the commons was clearly represented in our model by the progression of NONE, CONTROL, and SINGLE. Because antibiotic use in one-drug treatment creates unconstrained directional selection for stronger resistance, the tragedy never ends. On the other hand, if directional selection is stopped by the tradeoff constraints in a two-drug treatment, equilibrium conditions arise in which there is no conflict between the individual and the public. In our two-drug treatments, the same conditions maximized individual health by speeding recovery and public health by increasing the frequency of uninfected patients. Thus, the tragedy of the commons need not constrain all rational strategies of antibiotic use.

Because COCKTAIL yielded the best results, the question arises as to what are the biological bases of Equations 7 and 8. We can suggest one scenario based on the Y model of tradeoffs [39]. Let resistance to both drugs A and B result from the activity of two independent efflux pumps that are fueled by a shared and limiting pool of ATP. If a mutation increases *MICA* by directing more energy to pump A, the pleiotropic consequences are that *MICB* is decreased because pump B is slowed. Thus, the shared and limiting pool of ATP explains the tradeoff. Linear and non-linear properties in the tradeoff lead to the different values of *ω*. We imagine that both pumps and their stated properties operate in both the *combined* and *separate* cocktail equations. The difference between the equations emerges in the action of the drugs that are not pumped out of the bacterial cell. If drugs A and B are sufficiently similar such that they attack the same cellular or metabolic process (e.g., DNA translation pathway), the single equation in *combined* is justified. This formulation is therefore an approximation of antagonistic or suppressive combinations [28]. On the other hand, if drugs A and B are sufficiently different such that they attack distinct pathways, they can inflict double damage and two equations are required as in the *separate* equation. This formation serves as an approximation for additive drug combinations. Because there is evidence that synergistic drug combinations increase the strength and frequency of multi-resistant mutants [34]–[36], our model is limited to the *separate* and *combined* cocktail strategies.

Although we invoke efflux pumps, ATP pools, and targeted metabolic pathways, we accept that other scenarios are possible. However, we hope that our interpretation offers a first step in developing a conceptual framework for our model. A conceptual guidance could help the search for drug pairs to be used in experimental cocktails. Although much is known about the metabolic and genetic basis of drug resistance, our understanding of how tradeoffs and pleiotropy may constraint multiple-drug resistance remains limited. Are additive drug pairs, exemplified by the *separate* equation, more likely to be realized than antagonistic or suppressive pairs expressed by the *combined* equation? It is our hope that our model will stimulate the needed conceptual and experimental explorations to answer such questions.

A final and critical question is whether the tradeoffs we require are possible and general. They have clearly been identified in clinically relevant pathogens [14]–[20], [27]–[37], [41], [42]. Evolutionary and ecological tradeoffs are often identified as genetic co-variances and have been well documented in a wide range of organisms [40]. If tradeoffs are general, the results of our model could be applied to control more than just bacteria in clinical settings. Plants and pest insects or pathogenic fungi offer another system in which trials could be readily implemented. Of course, it remains to be determined if the constraints of tradeoffs can be broken by long-term evolution. However, unless evolutionary and ecological biology has oversold the importance of tradeoffs, it should be possible to use tradeoffs to our advantage in combating the evolution of antibiotic resistance, at least in the short term.

## Supporting Information

Table S1 Drug Susceptibility Conditions. Bacterial growth depends powerfully on a strain’s Minimum Inhibitory Concentration (MIC) in relation to the concentration of the drug used during treatment. The exploration of our model, and all associated figures, use the schema shown in Table S1. Where max ΔMIC denotes the difference between the most susceptible and most resistant strains.
(DOCX)

Table S2 Alternate Drug Susceptibility Conditions. To ensure that our exploration of MIC parameter space does not determine the model outcomes, we compared the results shown in Table S1 to a second resistance schema. Note that the difference between the most susceptible and the most resistant strain is much smaller (Table S2).
(DOCX)

Table S3Absolute Difference in Proportion of Uninfected Individuals. We found that the largest change in 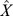 occurs when *ω* approaches 1. As *ω* decreases beyond the treatment-specific tradeoff threshold, the difference between the two schemas becomes vanishingly small. Absolute differences between schema-outcomes before, and after, the tradeoff threshold are shown in Table S3.(DOCX)

File S1 (DOC)
