# Supplementary material for: A Population Model Evaluating the Consequences of the Evolution of Double-Resistance and Tradeoffs on the Benefits of Two-Drug Antibiotic Treatments
Source: PLoS One. 2014 Jan 31;9(1):e86971. doi: 10.1371/journal.pone.0086971 (PMC3909004; doi:10.1371/journal.pone.0086971)
Supplement: Table S1 — Drug Susceptibility Conditions. Bacterial growth depends powerfully on a strain’s Minimum Inhibitory Concentration (MIC) in relation to the concentration of the drug used during treatment. The exploration of our model, and all associated figures, use the schema shown in Table S1. Where max ΔMIC denotes the difference between the most susceptible and most resistant strains. (DOCX) [file pone.0086971.s001.docx]

**Table S1**

| **MIC** | **S** | **R1** | **R2** | **R3** | **max ΔMIC** |
| --- | --- | --- | --- | --- | --- |
| Drug A | 0.000001 | 240 | 0.000001 | 120 * *ω* | 2.4 * 10^9^ |
| Drug B | 0.000001 | 0.000001 | 240 | 120 * *ω* | 2.4 * 10^9^ |
