# Supplementary material for: A Population Model Evaluating the Consequences of the Evolution of Double-Resistance and Tradeoffs on the Benefits of Two-Drug Antibiotic Treatments
Source: PLoS One. 2014 Jan 31;9(1):e86971. doi: 10.1371/journal.pone.0086971 (PMC3909004; doi:10.1371/journal.pone.0086971)
Supplement: Table S2 — Alternate Drug Susceptibility Conditions. To ensure that our exploration of MIC parameter space does not determine the model outcomes, we compared the results shown in Table S1 to a second resistance schema. Note that the difference between the most susceptible and the most resistant strain is much smaller (Table S2). (DOCX) [file pone.0086971.s002.docx]

**Table S2**

| **MIC** | **S** | **R1** | **R2** | **R3** | **max** **ΔMIC** |
| --- | --- | --- | --- | --- | --- |
| Drug A | 2.4 | 240 | 2.4 | 120 * *ω* | 1.0 * 10^2^ |
| Drug B | 2.4 | 2.4 | 240 | 120 * *ω* | 1.0 * 10^2^ |
