# Supplementary figures and images for: A Population Model Evaluating the Consequences of the Evolution of Double-Resistance and Tradeoffs on the Benefits of Two-Drug Antibiotic Treatments
Source: PLoS One. 2014 Jan 31;9(1):e86971. doi: 10.1371/journal.pone.0086971 (PMC3909004; doi:10.1371/journal.pone.0086971)

**Table S3**

| | | %Δ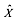| | | --- | | **Combined** | **Separate** | **Mix** | **Cycle** |
| --- | --- | --- | --- | --- | --- |
| ***ω > threshold*** | 4.95* 10-5 | 1.01 * 10-4 | 3.21* 10-5 | 3.17* 10-5 |
| ***ω < threshold*** | 5.32 * 10-12 | 1.11* 10-11 | 1.49* 10-10 | 4.46* 10-11 |

Supplement: Table S3 — Absolute Difference in Proportion of Uninfected Individuals. We found that the largest change in occurs when ω approaches 1. As ω decreases beyond the treatment-specific tradeoff threshold, the difference between the two schemas becomes vanishingly small. Absolute differences between schema-outcomes before, and after, the tradeoff threshold are shown in Table S3. (DOCX) [file pone.0086971.s003.docx]
