# Supplementary material for: A Population Model Evaluating the Consequences of the Evolution of Double-Resistance and Tradeoffs on the Benefits of Two-Drug Antibiotic Treatments
Source: PLoS One. 2014 Jan 31;9(1):e86971. doi: 10.1371/journal.pone.0086971 (PMC3909004; doi:10.1371/journal.pone.0086971)
Supplement: File S1 — (DOC) [file pone.0086971.s004.doc]

**File S1**

**Analytical Solutions**

**NONE**

With no antibiotics, (Equations 1–4) because *S* bacteria do not pay a cost *c*. As a result, *S* increases to a non-zero equilibrium frequency of $\hat{S}$, while the other infected patients decline to equilibrium frequencies of . To obtain $\hat{X}$, let = 0, or

$$= (G_{S} - \gamma) + \beta\hat{X}$$

$\hat{X} = -(G_{S} - \gamma)/\beta$ (S1)

Because *G_S_* = *φ*_max_ with no antibiotics (*a* = 0; Equation 6), Equation S1 becomes

$\hat{X} = -(\phi_{\max} - \gamma)/\beta$ (S2)

With the standard parameter values, $\hat{X}$= 0%.

**CONTROL**

With only antibiotic A and no resistant mutants, $R_{1}$=$R_{2}$=$R_{3}$= 0. Equation S1 offers again the solution for $\hat{X}$, except that the presence of the drug lowers *G_S_* as described by Equation 6. For *a* = 240 and the standard parameter values, *G_S_* = – 0.25, and $\hat{X}$= 50%.

**SINGLE**

With antibiotic A and the presence of resistant mutants, $\hat{R}_{1}$achieves to a value greater-than-zero. because *S*, *R*_2_ and *R*_3_ are more sensitive to the drug. $\hat{X}$is obtained by setting = 0 (Equation 2), or

$$= (G_{1} - \gamma) + \beta\hat{X} (1 - c)$$

$\hat{X} = \frac{-(G_{1} - \gamma)}{\beta(1-c)}$ (S3)

With the standard parameter values, *G*_1_ = 0 (Equation 6) and $\hat{X}$= 27.8%.

**Combined COCKTAIL**

The concentrations *a* = *b* = 120 in our cocktails are sufficiently high that *G*_s_ = *G*_1_ = *G*_2_ = *φ*_min_ = –.25 (Equation 7). As *ω* is increased from 0→1, the value of *G*_3_ = 0 → *φ*_min_. As a result, if *ω* is below a threshold *θ*, *R*_3_ dominates,$\hat{R}_{3}$> 0 and . If *ω* is above the threshold, *G*_s_ = *G*_1_ = *G*_2_ ≈ *G*_3_ and *S* dominates because it is the only one that does not pay the cost *c*.

The value of $\hat{R}_{3}$below *θ* is obtained by setting = 0 (Equation 4), or

$$= (G_{3} - \gamma) + \beta X (1 - c)$$

$\hat{X} = \frac{-(G_{3} - \gamma)}{\beta(1 - c)}$ (S4)

With *ω* = 0, *a* = 120, *b* = 120, and the standard parameter values, *G*_3_ = 0 (Equation 7) and $\hat{X}$= 27.8%. If 0 < *ω* < *θ*, Equation S4 is still valid but a new value of *G*_3_ needs to be determined for each value of *ω*.

The value of $\hat{X}$for *ω* > *θ* is given by Equation S1 because $R_{1}$=$R_{2}$=$R_{3}$= 0. However, with the presence of antibiotics *G_S_* = *φ*_min_ = –.25 (see above) and $\hat{X}$= 50%, as it was in CONTROL. This solution for $\hat{X}$holds for all values of *ω* > *θ* because *G_S_* is not affected by *ω*.

The threshold is obtained by noting that $\hat{S}$=$\hat{R}_{1}$=$\hat{R}_{2}$= 0 and *R*_3_ and *X* are the only types of patients present when *ω* < *θ*. Thus, $\hat{R}_{3}$+$\hat{X}$= 1. As *ω* is increased, the threshold is crossed when *S* is able to invade and > 0, or from Equation 1

$$< (G_{S} - \gamma) + \beta\hat{X} + \sigma\beta c(1-\hat{X}$$

$\hat{X} < \frac{-(G_{S} - \gamma)-\sigma\beta c}{\beta(1-\sigma c)}$ (S5)

By combining Equations S4 and S5 and rearranging, the conditions needed for *S* to invade as *ω* is increased becomes

$G_{3} < \frac{(1-c)(G_{S} - \gamma+\sigma\beta c)}{(1-\sigma c)}+ \gamma$ (S6)

With *G_S_* = *φ*_min_ = –.25 and the standard parameter values,

$$G_{3} < 0.1885$$

With *a* = 120 and *b* = 120 in Equation 7, *G*_3_ ≤ 0.1885 when *ω* ≥ 0.8597, which matches well the numerical estimate *θ* = 0.86 for Combined COCKTAIL in Figure 2.

**Separate COCKTAIL**

A threshold *θ* also exists in Separate COCKTAIL. Our numerical results had shown that *R*_3_ is the only bacterium with a greater than zero equilibrium when *ω* < *θ*. *R*_1_ and *R*_2_ are the only ones when *ω* > *θ*.

If *ω* < *θ*, $\hat{X}$is given by Equation S4 because *S*, *R*_1_, and *R*_2_ are not present. With *ω* = 0, *a* = 120, *b* = 120, *MICA*_3_ = 240, *MICB*_3_ = 240, and the standard parameter values, and *G*_3_ = – 0.0833 (Equation 8), and $\hat{X}$= 37.0%. Equation S4 can be used for other values of *ω* < *θ*, but a new value of *G*_3_ must be determined each time.

If *ω* > *θ*, $\hat{X}$is given by Equation S3. For *a* = 120, *b* = 120, and the standard parameter values, *G*_1_ = – 0.4167 (Equation 8) and $\hat{X}$=74.1%. Because *G*_1_ is not affected by *ω*, this solution for $\hat{X}$is valid for all values of *ω* > *θ*.

The threshold is obtained by starting with *ω* < *θ* and *R*_3_ as the only bacterium present. If *ω* is increased, there will be a value at which *R*_1_ and *R*_2_ first invade. Noting that $\hat{S}$= 0 and that *R*_1_ invades when > 0 (Equation 2)

$< (G_{1} - \gamma) + \beta\hat{X} (1 - c)$ (S7)

Combining Equation S7 and S4 through $\hat{X}$and rearranging, *R*_1_ is able to invade as *ω* is increased if

$$G_{1} > G_{3}$$

Because *G*_1_ is not affected by *ω*, its value is fixed at *G*_1_ = – 0.4167 (see above). By varying *ω* in Equation 8 with the standard parameter values, *G*_3_ decreases to – 0.4167 when *ω* is increased to 0.75, which matches exactly the threshold identified numerically for Separate COCKTAIL in Figure 2.
